# Intersectionality and Gender-Based Violence: An Empirical Multi-Level Examination of Prevalence and Frequency in Universities and Research Organizations

**DOI:** 10.1177/10778012241265363

**Published:** 2024-07-23

**Authors:** Anne Laure Humbert, Sofia Strid

**Affiliations:** 1 Centre for Diversity Policy Research and Practice, 6395 Oxford Brookes University, Oxford, UK; 2 Department of Sociology and Work Science, 3570 University of Gothenburg, Gothenburg, Sweden

**Keywords:** gender-based violence, intersectionality, MAIHDA, research organizations, universities

## Abstract

This article provides a multilevel intersectional analysis of the prevalence and frequency of gender-based violence within universities and other research organizations in Europe. Results show not only the high prevalence of gender-based violence in this context, but also that in contrast to the prevailing discourse, that gender-based violence is not solely a “women's problem”, but also a structural issue impacting diverse identities. Data on frequency show that physical and sexual violence usually occurs as isolated incidents, whereas psychological violence and harassment are often repeated.

## Introduction

Across Europe, numerous universities have appeared in the media with reports of gender-based violence as rife within their midst. While gender-based violence can take several forms, the extent to which rape culture or sexual harassment affected academic communities has become prominent. In the UK, for example, the website platform “Everyone's invited” launched a repository of testimonies in the first half of 2020. Originally tackling experiences of violence in schools, and particularly private schools, the testimonies soon encompassed experiences of students in different universities. Similarly, the #MeTooPhD movement revealed how widespread sexual harassment and assault were among university staff and students. While it appears to be well-established that gender-based violence is widespread and systemic in higher education and research, with severe negative consequences for institutions (economic, reputational), individuals (health and wellbeing, and relatedly study and career outcomes) and societies (Anitha & Lewis, 2018; Bondestam & Lundqvist, 2020), there has been to date little measurement of the scale of the problem. This is despite the measurement of gender-based violence being a prerequisite for the design, deployment, and implementation of effective institutional measures to combat and ultimately, eradicate gender-based violence in universities and research organizations (Humbert et al., 2024).

Examining gender-based violence in the context of universities and other research organizations has never been so timely. At the EU level, the policy priorities of the European Research Area (European Commission, 2012) did not focus on gender-based violence when first unveiled in 2012. However, with the Council Conclusions on the new ERA for Research and Innovation (R&I) (13567/20) and the European Commission Communication on the new ERA (COM(2020) 628 final), the need to address gender-based discrimination and violence in R&I organizations became explicit. By 2021, as part of efforts to work towards “deepening the ERA” (Council of the European Union, 2021b), ERA Action 5 sought to “Promote gender equality and foster inclusiveness, taking note of the Ljubljana Declaration” (Council of the European Union, 2021a). Gender-based violence is specifically addressed as an outcome of ERA Action 5: “Strategy to counteract gender-based violence including sexual harassment in the European R&I system and to assure gender equality in working environments through institutional change in any research funding or performing organization” of the ERA Policy Agenda 2020–2024. In 2022 the EU Member States were invited to express their commitment to specific ERA Actions and for Action 5, including “Working towards the co-development of an EU baseline strategic engagement, such as an EU zero tolerance code of conduct, on gender-based violence in R&I”. Gender-based violence also became one of the five recommended priority areas to be tackled by RPOs under the Gender Equality Plan (GEP) eligibility criterion for EU research funding (European Commission, 2021).

The purpose of this article is thus to provide empirical evidence on gender-based violence in higher education and research. It is based on the largest cross-cultural survey of its kind in Europe, including 46 research performing organization across 15 countries carried out between January 2022 and May 2022 (Lipinsky et al., 2022). The survey was conducted as part of the Horizon 2020 funded project UniSAFE (https://unisafe-gbv.eu/). These data produce evidence of the scale of the problem, not only among women but also among men and people who identify as non-binary or with another gender identity. It provides estimates of the prevalence of gender-based violence and its different forms, across staff and students, as well as data about its frequency. The analysis does, however, go further than presenting empirical data; it provides new insights by applying multi-level intersectional modeling to examine in depth how different identities and sets of social relations affect experiences of gender-based violence in the context of universities. This allows for a modeling of differences between groups, but also considers the effects of intersectional identities. Doing so allows us to establish how different identities and sets of social relations affect experiences of gender-based violence at the individual and structural levels. This new knowledge, in turn, aims at informing inclusive policy and practice at both the national and institutional levels.

## Gender-Based Violence and Intersectionality

Gender-based violence is an intricate issue associated with grave, and at times deadly, consequences. If violence appears to be universal, there is however a danger in regarding it as a-historic, identity neutral, and a-contextual (Colpitts, 2022). It is thus impossible to address this problem without considering its relationship with wider contexts and systems of inequalities (Heise, 1998; Humbert et al., 2024; Walby, 2009; Wemrell et al., 2019). Violence embodies part of a broader system of dominance and power inequalities, going beyond a binary understanding of gender and narrow legalistic definitions of gender-based violence (Humbert et al., 2024; O’Connor et al., 2021; Strid et al., 2021). Within this framing, gender-based violence is related to power, but not reducible to power; it is an expression of power rather than an expression of the lack of power (Walby et al., 2012). This perspective is particularly pertinent for examining gender-based violence within academia, as its distinct organizational attributes—such as gender-biased and hierarchical structures, cultures promoting gender inequalities, neoliberal cultures with unhealthy competition for publications and funding, and a significant reliance on precarious, temporary, and internationally mobile employment forms—may amplify the problem of gender-based violence (Bondestam & Lundqvist, 2020; Naezer et al., 2019; O’Connor et al., 2021).

Gender-based violence takes many different forms; it includes physical violence, psychological violence, sexual violence, and economic violence, and happens in both online and offline contexts (Council of Europe, 2018). It has different meanings in different national organizational contexts. While recognizing the potential conflicts and tensions emanating from different strands of epistemology, ontology, and contexts, the understanding here is primarily informed by a feminist and intersectional understanding of violence. In such understanding, gender-based violence: is a cause and consequence of gender inequalities and an inequality in its own right (Hearn et al., 2022); an expression of power and structural dominance, rather than as an expression of the loss of power and individual marginalization; analyzed as directed from the relatively privileged and powerful and directed towards the relatively disadvantaged (Humbert et al., 2024); includes a continuum of violence and violations, violent behaviors and attitudes based on sex and gender (Kelly, 1988); and always intersects with and mutually shapes other dimensions of inequalities, such as for example age, class, ethnicity, disability, and sexuality (Walby et al., 2012).

Gender-based violence transcends the confines of violence directed exclusively at women and permeates across all gender identities, notwithstanding the fact that women and minoritized groups are affected disproportionately. While the majority of gender-based violence incidents involve men's perpetration against women, and the intensity and consequences of violence women suffer at the hands of men are notably more severe, it is important to note that not all instances of gender-based violence are instigated by men or directed towards women. Consequently, although quantifying gender-based violence against women is an essential preliminary step, expanding this measurement to include gender-based violence that impacts other gender groups is critically important. This is particularly significant as the risk of exposure to gender-based violence is comparatively higher for non-binary and trans individuals (Davidson, 2016; European Union Agency for Fundamental Rights, 2020; Humbert et al., 2024; Jordan et al., 2020; Voth Schrag, 2017). When the most minoritized groups are left invisible and their voices largely silenced, this could also be seen as a form of epistemic violence (Banerjee and Hwang, 2023, Pérez, 2019). Furthermore, some instances of gender-based violence do occur against men, but these incidents are often suppressed and heavily stigmatized (Thobejane et al., 2018).

Gender needs to be seen as mediated through other identities, such as race/ethnicity or sexual orientation (Banerjee and Hwang, 2023). Membership of specific minoritized groups has been linked to an increased prevalence of gender-based violence, with patterns from the wider societal context echoed within the academic environment of universities and other research organizations. These groups encompass diverse identities such as gender identity (Humbert et al., 2024); sexual orientation minorities (Messinger, 2011); ethnic, racial, or cultural minorities (Roudsari et al., 2009; Wemrell et al., 2019); migrants (Gonçalves & Matos, 2020; Keygnaert & Guieu, 2015; Voolma, 2018); younger people (Voth Schrag, 2017), international students (Forbes-Mewett & McCulloch, 2016), and those engaged in precarious employment contracts (Bondestam & Lundqvist, 2020), among others. However, to date, there are few sources of systematic quantitative data on the prevalence of gender-based violence and its different forms, specifically within the context of universities that allow for an in-depth analysis of differences across groups and from an intersectional perspective.

This paper responds to this gap by adopting an intersectional approach to examine gender-based violence. Intersectionality can be both theoretical and empirical (Banerjee and Hwang, 2023) and in this article, is understood as a stance to shed light on the experiences of different groups, particularly the most minoritized ones, and then implemented in a multi-level intersectional approach to derive empirical results. However, we stress the importance of not losing sight of the political aim of intersectionality, seeing our work here and beyond as informing and sustaining a more profound change in institutions and addressing the problem of gender-based violence, by translating them into actions that address structural systems of inequalities (Colpitts, 2022; Humbert et al., 2024).

Intersectionality acknowledges that various forms of inequalities are influenced by different power dynamics within diverse social relations (Walby et al., 2012). Despite the growing recognition of intersectionality in both research and policy contexts, there is a dearth of empirical studies on gender-based violence that explicitly address intersectionality (Musso et al., 2020). By employing an intersectional analytical approach, we can expand our understanding of gender-based violence beyond the experiences of women alone. We are particularly mindful of the tendency for research to mostly focus on the experiences of white, cis-gender women, reflecting the perspective of what is presented at the “ideal” survivor (Colpitts, 2022) and leaving the assumptions behind the conflation of “violence against women” and “gender-based violence” unquestioned. An intersectional approach therefore allows for the disaggregation of gender-based violence experiences according to various groups, considering factors that may contribute to disadvantages, vulnerabilities, and differential consequences.

However, the categorization of discrete “groups” or “categories” itself presents challenges, as there is a risk of essentializing and reifying differences or perpetuating notions of uniformity within these groups (Crenshaw, 1991; McCall, 2005; Walby et al., 2012). One way to move beyond the debate over the fluidity or stability of categories is to view them as “heuristic devices” (Cho et al., 2013, p. 786), balancing the need for stable analytical categories with the recognition of their fluidity (Walby et al., 2012). Such an approach allows us to shift the focus from “categories of identity” towards understanding the “structure of inequalities” (Cho et al., 2013, p. 797) and to regard intersectionality as an “analytical sensibility” (Cho et al., 2013, p. 795). As we have previously argued (Humbert et al., 2024), a way forward is to follow the advice of Walby and colleagues (2012, p. 231) who stress the need to:

recognize the historically constructed nature of social inequalities and their sedimentations in social institutions […]. At any one moment in time, these relations of inequality have some stability as a consequence of their institutionalization, but over a period of time they do change. The institutionalization of social relations often provides a degree of relative stability to the experience of social inequality.

In this article, this means regarding certain social groups are systematically disadvantaged the dominance structure of inequalities, and analyzing variations in the experiences of gender-based violence that arise across different contexts (Humbert et al., 2024).

By embedding intersectionality into the analytical approach, we go beyond a unidimensional, additive, account of experiences of gender-based violence by simultaneously incorporating the effect of intersectional stratum membership. This recognizes, in line with intersectionality theory, that there are effects above and beyond that of individual level identities (Bauer et al., 2021; Evans et al., 2018; Weldon, 2006). A growing body of scholarship, originally stemming from epidemiology, has developed an innovative approach to carry out quantitative analyses informed by intersectionality. This approach, often referred to as multi-level analysis of individual heterogeneity and discriminatory accuracy (MAIHDA) provides insights into differences between groups while also considering the sets of social relations at the heart of structural inequalities (Evans et al., 2018; Merlo, 2018). In this analysis, we translate our original concerns about the relevance of intersectionality theory for the prevalence and frequency of gender-based violence in universities and other research organizations. To do so, we now provide a more in-depth description of our methodological approach.

## Methodology

### ***Description of Surve*** y

The analysis draws upon a survey carried out as part of the Horizon 2020 funded UniSAFE project (https://unisafe-gbv.eu/) implemented in 46 universities and other research organizations across 15 European countries between January and May 2022. The survey is a quasi-census of all staff and students at these institutions and collected n = 42,186 responses (of which n = 17,967 staff and n = 24,193 students). The survey's focus was explicitly on gender-based violence in the context of universities and other research organizations. The survey starts from the definition of gender-based violence provided by the Istanbul Convention (Council of Europe, 2011) and which focuses on physical violence, psychological violence, sexual violence, and economic violence. In addition, it considers sexual harassment and online forms of violence as topics that are particularly relevant to the context of academic or research institutions. It also opts to measure gender-based violence beyond the experiences of women only. This recognizes the role of gender in creating an asymmetry in experiences of violence. It is grounded in an understanding that sees violence in itself as a regime of inequalities (Hearn et al., 2022). By extending the measurement to all genders, including people that do not conform to the gender binary, it then becomes possible to understand how experiences of violence relate to inequalities and the experiences of different groups.

Gender-based violence is measured by adapting the approach developed by the Conflict Tactics Scales (CTS) (Straus et al., 1996) to the context of research organizations. This approach measures the methods of violence used; that is, it focuses on asking about actual incidents without resorting to labels. For example, the use of the term “rape” has been shown to be problematic, as not all will recognize what happened to them as rape and/or because the definition will vary in different contexts (Kelly, 1988). The use of the CTS instead asks about incidents, with a sample item for measuring rape being; for example, whether someone was forced into sexual intercourse by being held down or hurt in some way. The full list of validated items is available from Humbert and colleagues (2022).

Prevalence was calculated as the weighted number of cases reporting at least one experience of violence divided by the total number of valid cases, multiplied by 100, i.e., all cases coded as “Prefer not to say” or “No answer: break-off” was excluded from the calculation. Prevalence was calculated individually for the specific forms of violence and for any form of gender-based violence. For any form of violence, cases were excluded from the calculation if there was no valid data for any of the specific forms of violence. The statistical coherence of the measurement framework, overall and across different forms of violence, was assessed first through reliability analysis and then through Confirmatory Factor Analysis (Humbert et al., 2022). Out of the 32 original items, 29 were retained.

Characteristics related to demographic and functional diversity are considered in the analysis. Demographic diversity includes variables such as ethnicity or age, while functional diversity examines organizational roles, such as occupation or contractual arrangements. The analysis is particularly interested in capturing the effects of different (intersectional) factors such as sexual orientation, gender identity, ethnicity, or international mobility, which may exacerbate exposure to the risk of violence. These factors are broad in scope, reflecting various aspects of socio-demographic and function aspects of diversity that are salient in the context of academia and research (Strid et al., 2021), and shape inequalities in this context.

### *Analytical Approach*

The analysis relies on intersectional multi-level modeling. This approach aims at reconciling the tenets of intersectionality theory with the requirements of a quantitative approach by temporarily stabilizing categories to enable empirical analysis (Walby et al., 2012) and relying on them as “heuristic devices” (Cho et al., 2013). This draws on scholarly work incorporating intersectional strata in multi-level models (Bauer et al., 2021; Evans et al., 2018; Merlo, 2018), making it possible to include all possible sets of intersections in the analysis in recognition of the fact that individuals are not independent of each other but may share similar experiences with other members of their intersectional membership group (Evans et al., 2018). Intersectional strata are generated according to seven variables: gender identity (4 possible responses); gender currently the same as sex assigned at birth (2); age groups in 5-year bands (10); staff type (2); disability or chronic illness (2); minority ethnic group (2); sexual orientation (6). This creates a total of 1,127 non-empty strata, ranging from 1 to 5,342 observations. The multi-level models are also able to account for how experiences might relate to the different organizations and countries in which people work and live.

We use a cross-classified multi-level model (Leckie, 2013) consisting of three levels: countries (level 4); organizations (level 3); and intersectional strata (level 2). For prevalence, as our dependent variables are dichotomous, we use a logit link function in a logistic model. For frequency, we fit an ordinal intersectional multi-level model. Frequency is measured in decreasing order (1: 6 times or more, 2: 2–5 times, 3: once), where once is used as the reference category. Since there are three ordinal categories, the model uses two log-odds contrasts. The first contrast compares the highest frequency (6 times or more) to the others (2–5 times, once). The second contrast compares repeated frequency (6 times or more, 2–5 times) to once. These contrasts thus examine frequencies from more severe to less severe. Proportional odds are assumed, i.e., that the effect of predictors will be the same across the two different contrasts.

We first fit an unadjusted random intercept model, i.e., a variance component model, with the corresponding intraclass correlations (ICCs) for the intersectional strata level providing a measure of variation at the level of the intersectional strata, while considering the context of organizations and countries. We use the threshold summarized by Axelsson Fisk et al. (2018) as a loose guide to interpret the magnitude of these ICCs: up to 1%—non-existent; 1% to <5%—poor; 5% to <10%—fair; 10% to <20%—good; 20% to 30%—very good; and above 30%—excellent. We then compute the intersectional interaction model, that is, incorporating additive terms for individual diversity characteristics. This results in a model with both additive and intersectional effects, where the ICCs can be interpreted as the remaining differences attributable to intersectionality, at least in relation to the set of additive variables used in the model. The ICCs are calculated on the assumption that prevalence and frequency represent a latent response, using the following formula: $ICC=is_{0}/(3.29+is_{0})$ . The models are fitted through the external software package “runmlwin” (Charlton et al., 2022; Leckie & Charlton, 2013) within Stata v17. We use the MCMC algorithm with a 500-iteration burn-in period followed by a monitoring period of 10,000 iterations, with initial values provided by the IGLS (PQL2 method) parameter estimates (Browne, 2022).

## Results

### *Prevalence of Gender-Based Violence*

**Figure 1.**
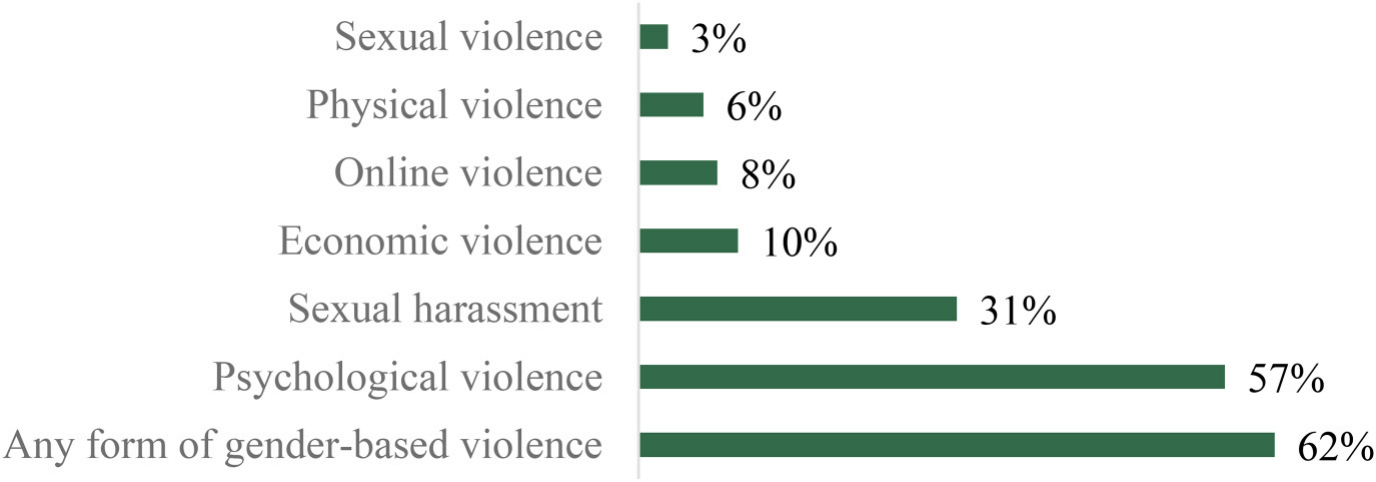
Prevalence of gender-based violence overall and by forms.

The results show the scale of the problem. Nearly two in three of the survey respondents, that is, 62%, report having experienced at least one form of gender-based violence in the context of their work or studies (Figure 1). Over half (57%) had experienced at least one incident of psychological violence, and nearly one in three (31%) at least one incident of sexual harassment. Other forms of gender-based violence were less prevalent, though even just 3% of respondents having experienced sexual violence still represents nearly 1,000 cases. Focusing on numbers of individuals rather than prevalence rates, our results show, for example, that there are approximately 12,000 respondents saying they had experienced sexual harassment.

*Source of data:* Lipinsky et al. (2022). Weighted estimates.

The analysis of prevalence using an intersectional multi-level approach (Table 1) provides an overview of the differences across groups that are attributable to individual identity categories and to membership of an intersectional stratum. The variance component models show ICCs at the intersectional strata level ranging from 5% to 23%, that is, from fair to very good (respective values are: any form of gender-based violence—10%; physical violence—7%; psychological violence—11%; economic violence—23%; sexual violence—16%; sexual harassment—10%; online violence—5%). This demonstrates that a large part of variation in the prevalence of gender-based violence can be attributed to belonging to different intersectional membership groups. After adding fixed effects to account for individual diversity characteristics, the corresponding ICCs for the intersectional strata level fall to between 1% and 4%, that is non-existent or at best poor (respective values are: any form of gender-based violence—2%; physical violence—1%; psychological violence—2%; economic violence—4%; sexual violence—1%; sexual harassment—2%; online violence—1%). From these results, we can conclude that differences in the prevalence of gender-based violence between intersectional identity groups are largely attributable to individual level characteristics, in an additive way.

**Table 1.** Intersectional Multi-Level Modeling of the Prevalence of Gender-Based Violence and Its Different Forms.

	Any form of gender-based violence		Physical violence		Psychological violence		Economic violence	
Constant	2.146***	1.741***	0.059***	0.028***	1.711***	1.351**	0.111***	0.133***
Student (Ref: Staff)		0.756***		1.644***		0.762***		0.475***
Women (Ref: Men)		1.619***		0.694***		1.584***		1.215**
Non-binary people (Ref: Men)		1.819***		0.793		1.452***		1.338
Sex at birth not aligned to current gender identity		1.173		1.541**		1.448***		0.689
Disability or chronic illness		1.643***		1.703***		1.589***		1.652***
Ethnic minority background		1.407***		1.684***		1.448***		2.092***
Asexual (Ref: Heterosexual)		1.053		0.975		0.958		1.096
Bisexual (Ref: Heterosexual)		1.475***		1.391***		1.404***		1.324***
Homosexual (Ref: Heterosexual)		1.414***		1.093		1.271***		1.123
Queer (Ref: Heterosexual)		1.759***		1.056		1.591***		1.440**
Another sexual orientation (Ref: Heterosexual)		1.341**		2.116***		1.384**		1.526**
International		0.976		0.899		1.039		1.276***
Age (mean-centered)		0.986***		0.979***		0.990***		1.015***
Time spent at the institution (mean-centered)		1.084***		1.058***		1.077***		1.042***
*v*_0_	0.100	0.060	0.052	0.070	0.091	0.050	0.302	0.213
*u*_0_	0.038	0.036	0.105	0.080	0.038	0.041	0.047	0.039
*is*_0_	0.382	0.071	0.266	0.031	0.392	0.075	1.006	0.148
Countries	15		15		15		15	
RPOs	46		46		46		46	
Intersectional strata	1,105		1,103		1,077		1,059	
Observations	38,095		38,049		36,459		33,631	
			Sexual violence		Sexual harassment		Online violence	
Constant			0.023***	0.002***	0.482***	0.306***	0.095***	0.072***
Student (Ref: Staff)				2.188***		0.815***		0.989
Women (Ref: Men)				2.004***		2.157***		0.944
Non-binary people (Ref: Men)				1.340		1.858***		1.029
Sex at birth not aligned to current gender identity				1.652*		1.247		1.112
Disability or chronic illness				1.688***		1.529***		1.701***
Ethnic minority background				1.460***		1.295***		1.597***
Asexual (Ref: Heterosexual)				0.800		1.285**		1.094
Bisexual (Ref: Heterosexual)				2.155***		1.665***		1.604***
Homosexual (Ref: Heterosexual)				1.456**		1.535***		1.363***
Queer (Ref: Heterosexual)				1.411		1.742***		1.693***
Another sexual orientation (Ref: Heterosexual)				1.863**		1.959***		1.797***
International				1.345*		0.953		0.922
Age (mean-centered)				0.950***		0.967***		0.999
Time spent at the institution (mean-centered)				1.097***		1.069***		1.031***
*v*_0_			0.070	0.075	0.122	0.069	0.016	0.013
*u*_0_			0.271	0.213	0.045	0.045	0.062	0.061
*is*_0_			0.633	0.045	0.379	0.076	0.156	0.022
Countries			15		15		15	
RPOs			46		46		46	
Intersectional strata			1,050		1,043		1,041	
Observations			32,909		32,502		32,040	

The intersectional interaction models show that students are overall less likely to experience gender-based violence in the context of their institutions (e^β ^= 0.756, *p* < .01), compared to staff. However, this does not apply evenly across different forms. Students are much more likely to experience physical violence (e^β ^= 1.644, *p* < .01) and sexual violence (e^β ^= 2.188, *p* < .01), but less likely to experience psychological violence (e^β ^= 0.762, *p* < .01) or sexual harassment (e^β ^= 0.815, *p* < .01). Compared with men, women are more likely to experience gender-based violence (e^β ^= 1.619, *p* < .01), including most of its forms such as psychological violence (e^β ^= 1.584, *p* < .01), sexual violence (e^β ^= 2.004, *p* < .01), or sexual harassment (e^β ^= 2.157, *p* < .01). However, it is people that identify as non-binary or another gender identity that appear to experience the most gender-based violence compared with men (e^β ^= 1.819, *p* <0.01), particularly psychological violence (e^β ^= 1.452, *p* < .01) and sexual harassment (e^β ^= 1.858, *p* < .01). While women were more likely to have experienced sexual violence, this does not apply to non-binary staff/students, showcasing how sexual violence against them does not reflect any embodied sexuality, but instead reflects gender harassment (Rabelo & Cortina, 2014). Trans respondents (i.e., sex at birth not aligned with current gender identity) were not more likely to experience gender-based violence overall, but were more likely to experience physical violence (e^β ^= 1.541, *p* < .05) and psychological violence (e^β ^= 1.448, *p* < .01).

Most sexual orientations other than heterosexuality— with the exception of asexuality—are associated with higher prevalence of gender-based violence overall (e^β^ range from 1.341 to 1.759, all with *p* < .01). Similarly to non-binary staff/students, increased prevalence is most pronounced for psychological violence and sexual harassment. In addition, minoritized sexual orientations are also associated with increased prevalence of online violence. It is also clear that both having a disability or chronic illness and coming from a minoritized ethnic background are associated with increased prevalence of gender-based violence overall (e^β ^= 1.643 and 1.404, respectively, both *p*s < .01), as well as across all the different forms of gender-based violence. Increased prevalence on economic violence was most marked (e^β ^= 2.092, *p* < .01) among staff/students from an ethnic minoritized background. Interestingly, contrary to expectations based on previous literature (Bondestam & Lundqvist, 2020; O’Connor et al., 2021), being an international staff/student was not associated with higher prevalence of gender-based violence, but only with higher prevalence of economic violence (e^β^ = 1.276, *p* < .01). Next, we examine the frequency of gender-based violence across different groups.

### *Frequency Across Different Groups*

**Figure 2.**
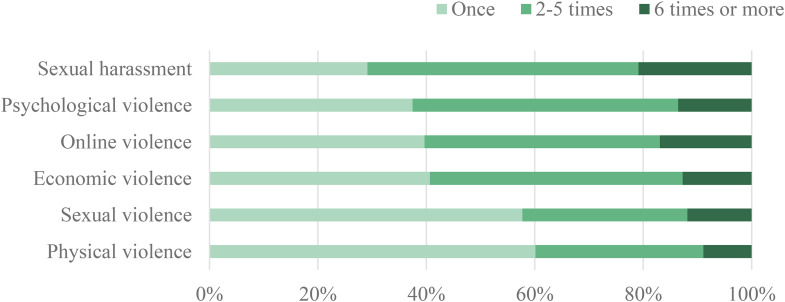
Frequency of Gender-based violence across different forms.

Looking at prevalence alone is not sufficient, and it also important to also look at the frequency of gender-based violence (Figure 2). The survey asked follow-up questions when respondents disclosed having experienced an incident. For incidents related to sexual violence or physical violence, the majority happened once (60% and 58%, respectively). In contrast, incidents related to sexual harassment or psychological violence were more likely to be repeated (71% and 63%, respectively).

*Source of data:* Lipinsky et al. (2022). Weighted estimates.

The use of ordinal intersectional multi-level modeling (Table 2) shows that for most forms of gender-based violence, there is little variation in the frequency of gender-based violence at the level of intersectional strata. The corresponding ICCs derived from the variance component models range from 0% to 9%, though all but one values are non-existent or poor (respective values are: physical violence—5%; psychological violence—4%; economic violence—5%; sexual violence—9%; sexual harassment—2%; online violence—2%). Adding fixed effects to account for individual diversity characteristics reduces these values even further, with most at or near 0% (respective values are: physical violence—3%; psychological violence—0%; economic violence—1%; sexual violence—0%; sexual harassment—0%; online violence—0%). These results suggest that not only is there little variation between groups in the frequency of gender-based violence experienced, but also that this is almost entirely accounted for by additive individual diversity characteristics, rather than intersectional strata.

**Table 2.** Intersectional Multi-Level Modelling of the Frequency of the Different Forms of Gender-Based Violence.

	Physical violence		Psychological violence		Economic violence	
Contrast 1: 6 times or more vs 2–5 times or once	0.073***	0.123***	0.195***	0.233***	0.130***	0.148***
Contrast 2: 6 times or more, 2–5 times vs once	0.652***	1.132	2.178***	2.624***	1.500***	1.733*
Student (Ref: Staff)		0.769		0.682***		0.754**
Women (Ref: Men)		0.689***		1.058		0.831**
Non-binary people (Ref: Men)		0.533		1.055		0.574
Sex at birth not aligned to current gender identity		1.464		1.295*		0.433*
Disability or chronic illness		1.660***		1.356***		1.265**
Ethnic minority background		1.331		1.249***		1.377**
Asexual (Ref: Heterosexual)		1.621		1.153		0.856
Bisexual (Ref: Heterosexual)		0.800		1.203***		0.895
Homosexual (Ref: Heterosexual)		0.780		0.944		0.845
Queer (Ref: Heterosexual)		0.960		1.095		1.504
Another sexual orientation (Ref: Heterosexual)		1.159		1.583***		1.443
International		1.023		1.089		1.107
Age (mean-centered)		0.999		1.005**		1.011*
Time spent at the institution (mean-centered)		0.978*		1.017***		1.011*
*v*0	0.074	0.056	0.037	0.037	0.125	0.122
*u*0	0.033	0.027	0.004	0.004	0.022	0.014
*is*0	0.172	0.114	0.134	0.011	0.181	0.029
Countries	15		15		15	
RPOs	44		46		46	
Intersectional strata	327		863		416	
Observations	1,778		20,409		3,696	
	Sexual violence		Sexual harassment		Online violence	
Contrast 1: 6 times or more vs 2–5 times or once	0.147***	0.272**	0.271***	0.435***	0.211***	0.213***
Contrast 2: 6 times or more, 2–5 times vs once	0.843	1.567	2.746***	4.445***	1.546***	1.583
Student (Ref: Staff)		0.652		0.846**		1.024
Women (Ref: Men)		0.717		0.740***		0.700***
Non-binary people (Ref: Men)		0.594		0.721*		0.714
Sex at birth not aligned to current gender identity		1.139		0.910		1.340
Disability or chronic illness		1.139		0.979		1.107
Ethnic minority background		1.596*		1.021		0.966
Asexual (Ref: Heterosexual)		1.300		1.008		0.851
Bisexual (Ref: Heterosexual)		0.987		1.098		1.173
Homosexual (Ref: Heterosexual)		1.005		0.858		1.332
Queer (Ref: Heterosexual)		0.870		1.050		1.194
Another sexual orientation (Ref: Heterosexual)		2.414*		1.738***		1.368
International		1.161		0.937		0.991
Age (mean-centered)		1.025		1.007*		1.014*
Time spent at the institution (mean-centered)		1.003		1.009**		1.005
*v*0	0.023	0.026	0.036	0.035	0.013	0.015
*u*0	0.017	0.031	0.015	0.016	0.010	0.008
*is*0	0.328	0.014	0.067	0.012	0.078	0.010
Countries	15		15		15	
RPOs	42		46		45	
Intersectional strata	191		675		377	
Observations	766		10,301		2,231	

The intersectional interaction models show some of the characteristics associated with higher frequency of gender-based violence. Women, compared with men, are less likely to experience repeat incidents of physical violence (e^β ^= 0.689, *p* < .01), economic violence (e^β ^= 0.831, *p* < .05), sexual harassment (e^β ^= 0.740, *p* < .05), or online violence (e^β ^= 0.700, *p* < .01). Students, compared to staff, are less likely to experience repeated incidents of psychological violence (e^β ^= 0.682, *p* < .01), economic violence (e^β ^= 0.754, *p* < .05), or sexual harassment (e^β ^= 0.846, *p* < .05). However, having a disability or chronic illness is associated with increased frequency of incidents of physical violence (e^β ^= 1.660, *p* < .01), psychological violence (e^β ^= 1.356, *p* < .01), and economic violence (e^β ^= 1.265, *p* < .05). Similarly, being from a minority ethnic background is also linked to increased frequency of psychological violence (e^β ^= 1.249, *p* < .01) and economic violence (e^β ^= 1.377, *p* < .05).

## Discussion and Conclusion

This paper provides empirical evidence on the prevalence and frequency of gender-based violence in the context of their institution across different groups, overall and across different forms (physical, psychological, economic, sexual, and online violence). By adopting an intersectional multilevel modeling approach, the paper highlights the complex interplay of structural inequalities that contribute to heightened vulnerability to violence among minoritized groups.

The data show that students are less likely to experience gender-based violence overall compared to staff, but they are more susceptible to physical and sexual violence. This might reflect differences in the context of interactions of staff and students. For example, students are potentially more exposed to situations where physical and sexual violence occurs, such as when socializing at parties or within intimate relationships formed with other students. Societal norms regarding gender roles might also reinforce the perception that (young) men ought to be aggressive and dominant, explaining higher rates of physical violence particularly among men students. The lower prevalence of gender-based violence among students can also be explained by differences in tenure. Students typically only spend up to 3 to 4 years in an institution, while staff members have longer tenures, which can increase their cumulative exposure to gender-based violence in the institution.

Women, in comparison to men, are more likely to experience gender-based violence in academic institutions, including psychological violence, sexual violence, and sexual harassment. Non-binary individuals experience the highest rate of gender-based violence, particularly psychological violence and sexual harassment. Trans respondents were not found to be more susceptible to gender-based violence overall, but they are more likely to experience physical and psychological violence. Those identifying with sexual orientations other than heterosexuality, with the exception of asexuality, are more likely to experience gender-based violence, especially psychological violence, sexual harassment, and online violence. These findings align with existing research that reflects deeply entrenched social norms and power imbalances that are both a cause and consequence of gender-based violence. These are particularly salient in light of data that show that those most at risk are those who most deviate from traditional “cis-het” gender norms. These findings therefore really speak to the need to not only focus on violence perpetrated against women, but to also consider how violence is deployed against other gender- or sexual-minoritized groups.

Individuals with a disability or chronic illness, or those from minoritized ethnic backgrounds, are also more likely to experience gender-based violence in the context of their work or study in academic institutions, with those from an ethnic minoritized background facing increased economic violence. People with disabilities or chronic illness may experience societal attitudes that stigmatize them and perceive them as less capable of defending themselves, which can be exploited by perpetrators. These results suggest that discrimination based on ableism is a definite factor in exacerbating experiences of gender-based violence. Similarly, discrimination in the form racism and xenophobia is deployed alongside sexism, resulting in higher prevalence of violence. These results underscore the importance of addressing intersecting forms of discrimination and inequalities in understanding and responding to gender-based violence.

Surprisingly, international students or staff did not show a higher prevalence of gender-based violence overall, only economic violence. This might be because international students and staff have stronger social support networks within institutions than domestic students and staff, whose support network might be more diffuse in the country of residence. These more concentrated networks may provide emotional support, practical assistance and resources that mitigate against the effects of not having a strong support network in the host country. It may also be that international students and staff face additional barriers to reporting, for reasons that include language barriers, fear of deportation for those staying under a visa, or not being familiar with local institutional regulations or legislation. The finding that international student and staff experience higher prevalence of economic violence may reflect greater precarity in living, working, and studying conditions, and greater reliance of financial resources in the absence of a support network in the country of residence.

In terms of frequency, incidents related to sexual violence or physical violence usually occur once, while incidents related to sexual harassment or psychological violence are more likely to be repeated. This could be related to how acute an incident is, i.e., a severe and immediate impact in terms of harm, more associated with sexual and physical violence, in contrast to an ongoing pattern of behavior that intends to intimidate, control, or degrade a person that are more associated with sexual harassment or psychological violence. Women, compared to men, are less likely to experience repeated incidents of various forms of violence. A possible explanation is that men may face societal norms and expectations that influence them to tolerate or ignore violence, particularly as violence is associated with masculinity and strength. However, those with a disability or chronic illness or from a minority ethnic background are more likely to face repeated incidents of physical, psychological, and economic violence. This is likely to reflect structural inequalities in access to resources and support networks, making them less likely to access inclusive support services, and thus isolating them and perpetuating cycles of violence.

These results challenge the prevailing discourse pertaining to gender-based violence, whether within research organizations or a broader societal context, predominantly propagates the misconception that it is exclusively a “women's problem”. This discourse often conflates gender-based violence with the more narrow topic of violence against women. Moreover, taking a narrow definition often means that women are not considered in any ontological depth. Instead, stereotypical assumptions dominate, dictating the “type of women” who are victims of gender-based violence. This often leans towards notions of vulnerability and powerlessness, or some form of “deficiency,” such as a migrant background. As we demonstrate, such portrayal is inconsistent with empirical findings. Gender-based violence transcends gender identities, impacting all individuals. Although women are demonstrated to have a higher prevalence compared with men, the highest prevalence rates of violence are observed among non-binary individuals. As previously argued (Humbert et al., 2024), this is attributed to societal backlash against those who deviate from the expected societal gender norms. Likewise, prevalence escalates in tandem with diverse factors, such as belonging to a minoritized group, or having a disability or chronic illness.

Our work therefore aligns with intersectionality theory, fully recognizing how diverse identities and the structural inequalities faced by different groups affect the prevalence and frequency of experiences of gender-based violence in an academic context. Our work underscores the importance of considering the complexity, contextuality, and multidimensionality of oppression in understanding and addressing violence. We do so not only theoretically but also methodologically. Analyses of gender-based violence can be conducted across different demographic groups based on these varying identity categories. However, this approach enables only an additive perspective, thereby obscuring the intersectional dynamics inherent in the prevalence of gender-based violence (Humbert, 2024; Spierings, 2012; Weldon, 2006). Transitioning from an additive to an intersectional multilevel modeling approach allows for shift of perspective—and consequently, interventions and measures—from an individual level to a structural one (Walby et al., 2012). This transition is also aligned with the transformative goals of intersectionality theory (May, 2015). In our analysis, we use intersectional strata as representative of social sets of relations comprised of intersections of different identity characteristics (Bauer et al., 2021; Evans et al., 2018; Merlo, 2018). These characteristics are chosen based on their significance in perpetuating minoritization and inequalities. By employing intersectional strata in our modeling, we integrate the principle of intersectionality at various levels of the ecological model (Heise, 1998; Humbert et al., 2024).

By engaging with critical feminist perspectives on gender-based violence, seeing violence as both a cause and consequence of gender inequalities and unequal power relations, our aim is to center around the experiences of violence of minoritized people, beyond the narrower framing of gender-based violence as affecting women only, and by disrupting this dominant narrative to stress the need for more inclusive and intersectional approaches to addressing gender-based violence. Empirical findings, such as the ones presented in this article, are indeed key in the process of working towards transformative justice and promoting institutional responses that address systemic inequalities and promote healing, accountability, and societal change. By centering the needs and experiences of minoritized groups, practitioners, policymakers, and advocates can work towards creating more inclusive, equitable, and responsive systems of support and justice for all victims and survivors.
